# Refutation of Dr. Hale's Opinions on Animal Heat

**Published:** 1814-10

**Authors:** B. C. Brodie


### *For the Medical and Physical Journal.*

### *Refutation of Dr. Hale's Opinions on Animal Heat; by* Mr. B. C. Brodie.

IN the last Number of your Journal, I see an account of an Inaugural Dissertation, by Dr. Hale, junior, in which the author states, that, having repeated some of my experiments on animal heat, he had met with very different results. This occasioned in me some surprise, as I had reason to believe that I had conducted my experiments with all that caution and attention to minute circumstances, which every physiologist knows to be absolutely necessary in such investigations, to enable us to arrive at any accurate conclusions. On perusing the article in question, however, I found that Dr. Hale in reality had not repeated my experiments, but had made others, bearing only a certain degree of analogy to them. The grounds of this assertion are, 1, that the whole of my comparative experiments were made on rabbits, and his appear to have been made on dogs; 2, that in Dr. Hale's experiments the brain was left undisturbed, except by the division of the spinal marrow, whereas, in mine, either the brain was removed, or its functions were completely suspended by means of the Woorara poison; 3, that Dr. Hale omitted to make the chemical examination of the respired air.

1. In all my comparative experiments, I was led to employ rabbits in preference to other animals, because it was more easy to procure them exactly of the same age, size, and colour (circumstances of great consequence to be attended to); and because in my second series of experiments they were of a convenient size for being included in the apparatus constructed for the examination of the respired air. When in a rabbit the circulation is maintained by means of artificial respiration after decapitation, the animal lies motionless, and exhibiting no signs of sensibility; whereas a dog under these circumstances is affected with violent convulsions, has repeated evacuations of fæces, and even seems to perform certain voluntary movements. It is not unreasonable to suppose, that, if in a dog the other animal functions continue to be performed in so great a degree, from the influence of the spinal marrow, after the brain is removed, from the same cause the faculty of generating heat should continue to exist in a slight degree also.

2. Dr. Hale, in simply dividing the spinal marrow, left an extensive nervous communication between the brain and the trunk, by means of the nerves situated in the neck. In two of his experiments, indeed, he states, that these nerves were divided; by which I conclude, that he made a section of the sympathetic nerves, and of those of the eighth pair, in the middle of the neck, since no others are accessible to an operation, nor these except in this situation. Even this, however, cannot be regarded as having been sufficient to interrupt the nervous communication between the brain and the rest of the system, since the nerves of the face, tongue, &c. and the anastomosing branches of the sympathetic, spinal, and other nerves in the upper part of the neck, were still entire, and capable of conveying the cerebral influence directly to some parts, and indirectly to other parts, of the body.

3. Dr. Hale, in neglecting to examine the respired air, omitted a very important part of the investigation. Had he found, as I did, that the animals in which the artificial breathing was employed consumed as much oxygen, and evolved as much carbonic acid, as under ordinary circumstances, he would have regarded his experiments as equally conclusive with mine against the chemical theory of animal heat.

Indeed this part of physiology still presents an ample field of inquiry. On the subject of animal heat we possess at present some negative, but very little positive, knowledge. The experiments of Dr. Hale are not without value; and I trust that he will continue to prosecute his researches. He will also, I am sure, excuse me if I take the liberty of suggesting, that, in the repetition of experiments made by others, too much attention cannot be paid to making them alike, even in the most minute particulars, as physiological science is not yet sufficiently advanced to enable us to pronounce with certainty what circumstances are, and what are not, of importance. B. C. BRODIE.

*Sept.* 4, 1814.

